# Post‐LECA Origin and Diversification of an Axonemal Outer Arm Dynein Motor

**DOI:** 10.1002/cm.70025

**Published:** 2025-08-08

**Authors:** Stephen M. King

**Affiliations:** ^1^ Department of Molecular Biology and Biophysics University of Connecticut Health Center Farmington Connecticut USA

**Keywords:** axoneme, cilia, dynein, microtubule, motor

## Abstract

Dyneins were present in the last eukaryotic common ancestor (LECA) and play key roles in eukaryotic biology. Axonemal dyneins form the inner and outer arms that power ciliary beating, and it has long been recognized that outer arms in some organisms contain two different heavy chain motors, whereas those from other species contain a third unit that imparts enhanced motive force during ciliary beating. Previous phylogenetic analyses suggested that this third motor derived from a gene duplication event in the LECA, followed by the subsequent replacement of the N‐terminal assembly domain with one formed from kelch and immunoglobulin repeats. Here I revisit the origin and organization of this dynein, combining the increased breadth of sequence information now available, AlphaFold modeling, and the recent recovery of a robustly rooted eukaryotic tree‐of‐life. This analysis confirms the third outer arm dynein HC arose in a common ancestor of the Diaphoretickes, with a basic N‐terminal domain consisting of a β‐propeller structure followed by two immunoglobulin folds. However, this region has undergone further diversification in some groups, gaining an additional full or partial β‐propeller located immediately adjacent to the AAA motor domain. Thus, three variant forms of this N‐terminal segment are discernable in extant eukaryotes.

**Abbreviations**
eTOL: eukaryotic tree‐of‐life
HC: heavy chain
IC: intermediate chain
Ig: immunoglobulin
LC: light chain
LECA: last eukaryotic common ancestor
LIC: light intermediate chain
SAR: Stramenopiles‐Alveolates‐Rhizaria

## 1 Introduction

Dyneins are microtubule‐based molecular motors that are present in most eukaryotic groups (Kollmar 2016; Wickstead 2018). Cytoplasmic dyneins generally act as the major microtubule minus end‐directed motor in cells; while a closely related dynein is needed for retrograde intraflagellar transport (IFT) in cilia. In addition, motile cilia contain a complex array of dyneins incorporated into the axonemal superstructure. These diverse motors generate the inter‐doublet microtubule sliding that underlies ciliary bend formation and propagation.

Dyneins are built around the heavy chain motor units that consist of an N‐terminal region involved in assembly, followed by a linker that spans across a ring of six dissimilar AAA domains (AAA1–AAA6). Nucleotide binding, hydrolysis, and product release at AAA1 drive dynein mechanochemistry; AAA2 contains but does not hydrolyze ATP, while AAA3 and AAA4 bind ATP or ADP and are involved in regulatory processes. The last two AAA domains do not associate with nucleotide but are important for structural transitions (Carter et al. 2011; Kon et al. 2012). The microtubule‐binding domain is located at the tip of an antiparallel coiled coil stalk that extends from AAA4, and changes in coil registry driven by the ATPase cycle in AAA1 control microtubule binding affinity. The AAA4‐derived stalk is supported and regulated by a second antiparallel coiled coil (the buttress) that comes from AAA5 (Carter et al. 2008; Chai et al. 2025).

Within cilia, there are three classes of axonemal dyneins: the outer dynein arms that provide most of the power output of the organelle and contain two or three different HCs depending on the source, the inner arm I1/f dynein that consists of a HC heterodimer needed for defining waveform, and a series of monomeric HC dyneins with varying motor properties that modify waveforms and are important for beating under specific conditions (King et al. 2023; Leung et al. 2025; Lin and Nicastro 2018; Yagi et al. 2005). Within each 96‐nm axonemal repeat unit, there are four outer arms, one inner arm I1/f, and six monomeric dyneins bound around the radial spokes that protrude towards the central pair apparatus (Nicastro et al. 2006). In some organisms such as the green alga *Chlamydomonas reinhardtii* , there are additional single HC dyneins that are present in low amounts, some of which are known to localize to restricted regions of the axoneme (Yagi et al. 2009). In addition to the HCs, each dynein type contains a series of other components that are required for holoenzyme formation, axonemal incorporation, motor function, and/or regulation (King 2018).

Cytoplasmic and IFT dyneins contain HC homodimers, while inner arm I1/f is a HC heterodimer and outer arms are either HC heterodimers or heterotrimers depending on the source. However, all multi‐HC dynein motors contain a core protein complement of two HCs that associate via a relatively well‐conserved dimerization interface that includes the DHC_1N domain and involves an intermediate chain/light chain (IC/LC) complex needed for holoenzyme assembly, as well as other motor‐specific components such as regulatory light chains or light intermediate chains (LICs) (King 2018).

Given the broad occurrence of dyneins across eukaryotic species, it is confidently predicted that cytoplasmic, IFT, and axonemal dynein motors were present in the last eukaryotic common ancestor (LECA) and that in general their absence in various extant organisms represents a history of gene loss in certain lineages (Wickstead 2018). For example, the yeast *Saccharomyces cerevisiae* has only canonical cytoplasmic dynein and lacks the IFT and all axonemal motors. In contrast, the chlorophyte *Chlamydomonas reinhardtii* has lost cytoplasmic dynein but retains a full complement of axonemal dyneins and the retrograde transport motor. Some organisms, such as conifers, angiosperms, and certain red algae, have lost all dyneins but instead must rely on an expanded repertoire of kinesins for microtubule minus end‐directed motility (Yamada et al. 2017). Thus, perhaps surprisingly, no one dynein type appears to be absolutely essential for all of eukaryotic life.

**FIGURE 1:**
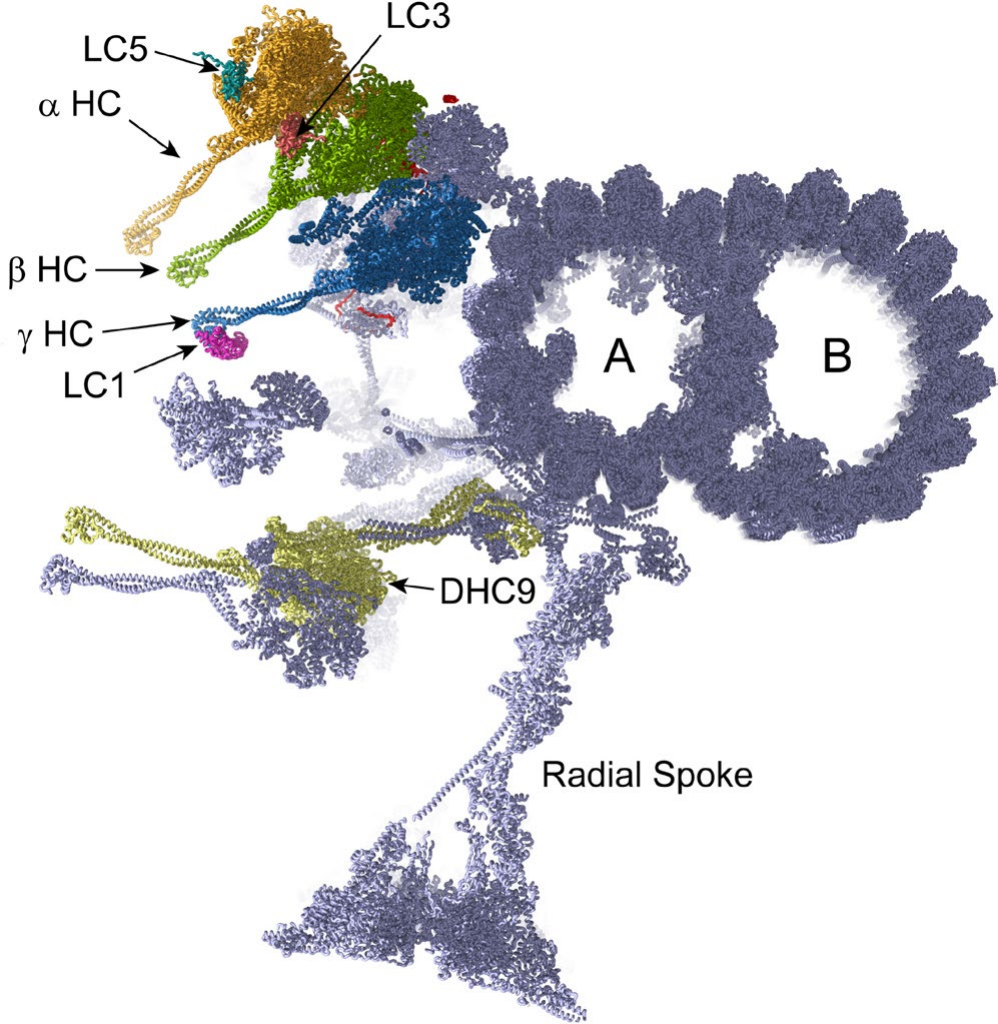
Arrangement of outer arm dynein heavy chains in situ. The α, β, and γ HCs of the *Chlamydomonas* outer dynein arm are colored orange, green, and blue, respectively. Orthologues of the β and γ HCs are found in all outer arms. However, the outermost HC motor has a much more restricted distribution, being found only in the Diaphoretickes. This third HC consists of an unusual N‐terminal domain required for association with the β HC, attached to a motor unit that is paralogous to that of the β HC. Also visible are the γ HC‐associated leucine‐rich repeat protein LC1 and the thioredoxin‐like LC3 and LC5 light chains that bind the β and α HCs, respectively. The DHC9 HC of inner arm dynein c is shown in yellow. This figure was prepared from the 96‐nm axonemal repeat cryo‐electron microscopy (EM) structure (PDB 8GLV; Walton et al. 2023).

However, it is also clear that some diversification of dynein motors has occurred since the LECA (Kollmar 2016). While most dyneins have a relatively well conserved protein complement, it has long been recognized that some organisms assemble outer arms that contain three HC motor units (Figure 1) while others have only two. These observations date back to the initial electrophoretic and electron microscopy studies of outer arm dyneins biochemically purified from multiple sources such as *Chlamydomonas* (Pfister et al. 1982), *Tetrahymena thermophila* (Johnson and Wall 1983), mammals (Hastie et al. 1986), fish (Gatti et al. 1989; King et al. 1997), sea urchins (Bell et al. 1979), sea squirts (Hozumi et al. 2006), and various other marine invertebrates, for example, (Mohri et al. 1999; Wada et al. 1992).

All outer arms contain orthologues of the *Chlamydomonas* β and γ HCs; however, the third HC found in some outer arms is quite distinct. Initial sequencing of this additional HC (termed α in *Chlamydomonas* but γ in *Tetrahymena*; see nomenclature note) by (Mitchell and Brown 1997) revealed that the AAA domain motor unit is closely related to (i.e., paralogous with) that of the β HC. However, the N‐terminal region appeared quite different from that of the other HCs (β and γ) from the same outer arm dynein and generally consists of a series of kelch repeats that form a β‐propeller structure often with six blades similar to those formed by WD‐repeats, followed by two immunoglobulin (Ig)‐like folds. It is now understood that, as the core HC‐HC dimerization interface between the β and γ HC orthologues, which also involves the IC/LC complex, is already fully occupied, this distinct N‐terminal domain allows for the association of the third outer arm motor at a site on the β HC distal to the standard HC‐HC dimerization interface (Rao et al. 2021; Walton et al. 2021). This interaction is very tight and, for example, in *Chlamydomonas*, extensive dialysis against a low ionic strength buffer is needed to disrupt it so that the individual HCs can be purified separately (Pfister and Witman 1984).

**FIGURE 2:**
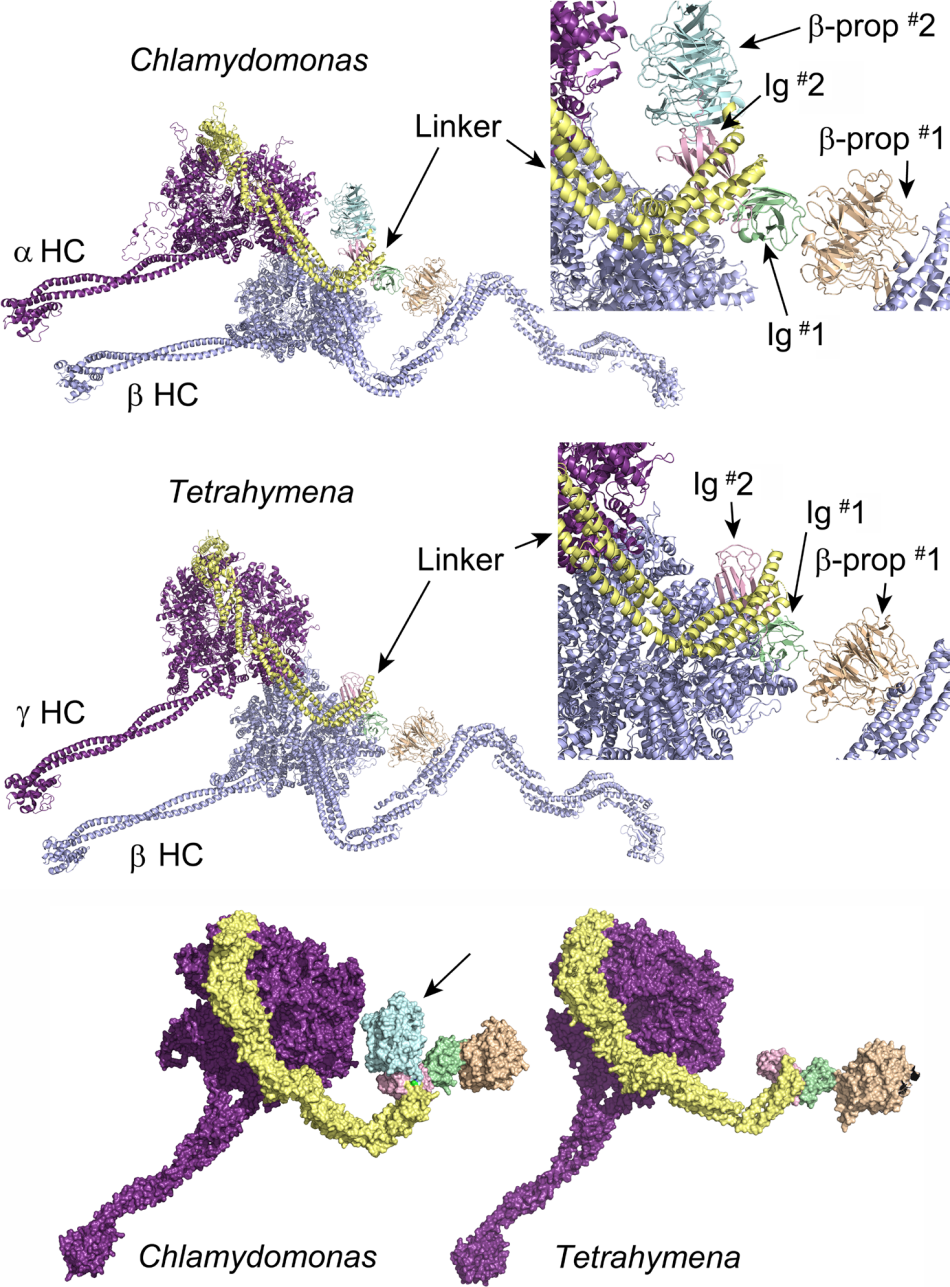
The outermost heavy chain of *Chlamydomonas* and *Tetrahymena* outer arm dyneins. The overall architecture of the *Chlamydomonas* and *Tetrahymena* outermost HCs is shown at *upper* and *middle left*—the motor domains are in violet‐purple, the linkers are yellow, and the N‐terminal regions are in wheat (β‐propeller ^#^1), pale green (Ig ^#^1), light pink (Ig ^#^2) and pale cyan (β‐propeller ^#^2). The β HCs are in light blue, and the innermost of the outer arm HCs have been omitted for clarity. Enlarged views of the ribbon structure of the N‐terminal regions are shown at *right*; the β‐propeller and Ig domains are indicated. Surface representations for the outermost HCs are in the *lower* panel; note that the *Chlamydomonas* N‐terminal domain contains an additional β‐propeller (arrow) that abuts the AAA ring. These views are from the experimental cryo‐EM structures 8GLV and 7K5B, respectively.

In a previous study, (Kollmar 2016) suggested that this third outer arm HC motor unit arose in the LECA as a duplication of the β HC and that the original N‐terminal domain was subsequently replaced in a common ancestor of the Diaphoretickes. There is, though, some controversy about when the original duplication occurred, as (Wickstead 2018) does not consider it feasible to accurately place this event. In recent years, enormous amounts of genomic and transcriptomic sequence information from a plethora of very divergent eukaryotic species have become available. This ever‐growing resource allows for the identification of enzymes consisting of a dynein AAA domain motor unit coupled to a kelch domain/Ig‐fold N‐terminal region that are predictably orthologous to the *Chlamydomonas* α HC and *Tetrahymena* γ HC (Figure 2). Thus, by looking for a β‐propeller motif contiguous with a dynein motor domain, it is feasible to search for potential orthologues of this extra outer arm dynein HC in an even broader array of eukaryotes than was previously possible. Thus, revisiting the original prediction is potentially worthwhile, as it might identify orthologues in other supergroups and/or confirm the previously proposed origin of this HC.

**FIGURE 3:**
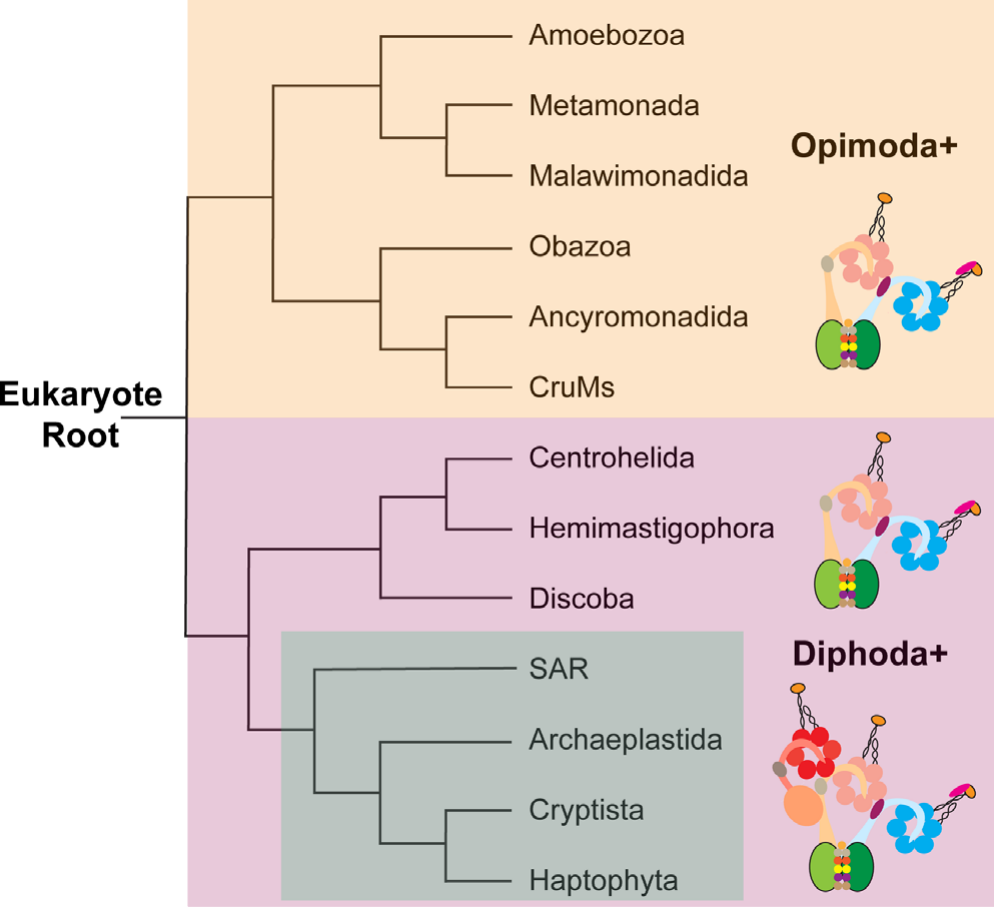
Eukaryotic phylogeny and origin of the third outer arm heavy chain. Phylogenetic tree illustrating the relationship between supergroups based on the study of Williamson et al. (2025). The root of the eukaryotes lies in the Excavates and splits the Discoba from the Metamonada and Malawimonadida. Members of the Opimoda+ (orange) build outer dynein arms containing two HC motor units. However, within Diphoda+ (pink), there is a dichotomy. Discoba have two‐headed outer arm dyneins, while those in SAR, Achaeplastida, Cryptista, and Haptophyta (together this combination is termed the Diaphoretickes (Adl et al. 2012); indicated by the green box) have three HCs. Thus, the most parsimonious interpretation is that this third HC arose in the Diphoda+ within a common ancestor of the Diaphoretickes following the split from Discoba and other lineages. For broader context, all Metazoans and Fungi are within the Obazoa. SAR represents the Stramenopila, Alveolata, and Rhizaria, and CRuMs refers to a microbial clade consisting of Collodictyonida, Rigifilida, and Mantamonadida. Due to the lack of available sequence data, it is not currently feasible to determine directly whether this third outer arm HC is present in the Hemimastigophora, which groups with and is sister to Discoba. However, a cross‐section electron micrograph of a cilium from the hemimastigophoran *Hemimastix amphikineta* shows an outer arm structure with a morphology consistent with two rather than three HCs (Foissner et al. 1988). The outer arm dynein diagrams are modified from (King 2021).

Although our understanding of the composition of eukaryotic supergroups and their inter‐relationships has undergone very considerable revision and refinement in the last several years, the root origin of eukaryotes and placement of the LECA root has remained somewhat uncertain and controversial (Al Jewari and Baldauf 2023; Burki et al. 2020). Now a very extensive phylogenomic analysis (Williamson et al. 2025) based on a large dataset of mitochondrial proteins from all currently recognized supergroups has recovered a eukaryotic tree‐of‐life (eTOL) that is robustly rooted and splits eukaryotes into two “multi‐supergroup assemblages” termed Opimoda+ and Diphoda+ (the “+” indicates that these new assemblages contain additional lineages from those originally included by (Derelle et al. 2015)). This study places the LECA root firmly within the polyphyletic group previously referred to as Excavates, which consists of Discoba (e.g., *Euglena*, *Trypanosoma*), Metamonada (e.g., *Giardia*, *Trichomonas*), and Malawimonadida (e.g., *Gefionella*, *Malawimonas*), splitting Discoba from the other two groups. When combined with the reordered placement of other supergroups, the new eTOL provides a comprehensive basis for interpreting organismal relationships (Figure 3).

Consequently, combining the new eTOL with more recently available sequences and AlphaFold models of dyneins from divergent eukaryotes, the origin of the third HC that occurs within some outer arm dyneins can be placed in the broader context of a rooted view of eukaryotic evolution. This analysis confirmed the occurrence of this dynein class in a Diaphoreticke ancestor. It also revealed that the unusual N‐terminal segment of this HC type underwent further modification and diversification following its first appearance, gaining an additional β‐propeller in some groups (as first noted in *Chlamydomonas* by Walton et al. 2023) and then, at least in one supergroup, partially losing it. Thus, as discussed below, three variant forms of this N‐terminal region are currently discernable in extant eukaryotes.

## 2 Occurrence and Predicted Origin of the Third Outer Arm Dynein Motor

The motor unit of this dynein HC is paralogous to that of the β HC; it has been proposed that this gene duplication occurred in the LECA (Kollmar 2016). Here, we are concerned with when that additional motor unit was first utilized as a third outer arm HC following the substitution of the original N‐terminal dimerization domain with the β‐propeller unit found in extant organisms. Similar to previous analyses, extensive searches of newly available sequences confirm that HCs with this unusual N‐terminal organization are present only in the Diaphoretickes (Table 1), which includes SAR, Cryptista, Haptophyta, and Archaeplastida. Orthologous sequences were not found in the Discoba, which forms the other major lineage within Diphoda+ or in any member of the Opimoda+. Previously, several HCs from the Heterolobosea (e.g., *Naegleria fowleri* ) which belong in the Discoba, were placed with the α HC group based on their motor domain sequences (Kollmar 2016). However, BLAST searches of the current NCBI database did not identify any heterolobosean dynein motor domain connected to an N‐terminal β‐propeller. Indeed, the *Naegleria* sequence (available at CyMoBase as NfDHC4A) contains a standard N‐terminal DHC_1N domain and so is not fully equivalent to the *Chlamydomonas* α HC. Indeed, it also lacks the enlarged heavily phosphorylated loop region from AAA5 that overlays the coiled coil buttress and microtubule‐binding stalk, which is another defining feature of the third outer arm HC (Sakato‐Antoku et al. 2025). Rather, this Naegleria dynein appears to represent a duplicated but otherwise undiversified β HC. Thus, there is no evidence that outer arms from Heterolobosea or Discoba more generally contain a third motor unit.

**TABLE 1:** Outer arm dynein heavy chains containing an N‐terminal β propeller domain.

Organism	Super group	Accession number
*Chlamydomonas reinhardtii*	Archaeplastida (Chlorophyta)	Cre03.g145127
*Chrysochromulina tobinii*	Haptophyta (Prymnesiophyceae)	KOO23494
*Diacronema lutheri*	Haptophyta (Pavlovophyceae)	KAG8462790
*Ectocarpus fasiculatus*	SAR (Stramenopila, Ochrophyta)	CAM9197700
*Guillardia theta*	Cryptista (Cryptophyta)	XP_005834820
*Micromonas pusilla*	Archaeplastida (Chlorophyta)	XP_003057702
*Phytophthora nicotianae*	SAR (Oomycota)	ETK96005
*Prymnesium parvum*	Haptophyta (Prymnesiophyceae)	KAL1521978
*Stentor coeruleus*	SAR (Alveolata, Heterotrichia)	OMJ82808
*Toxoplasma gondii*	SAR (Alveolata, Apicomplexa)	KAF4641141
*Tetrahymena thermophia*	SAR (Alveolata, Oligohymenophorea)	7K5B_C
*Triparma verrucosa*	SAR (Stramenopila, Ochrophyta)	GMH97409

## 3 Structural Diversification Within the Diaphoretickes

**FIGURE 4:**
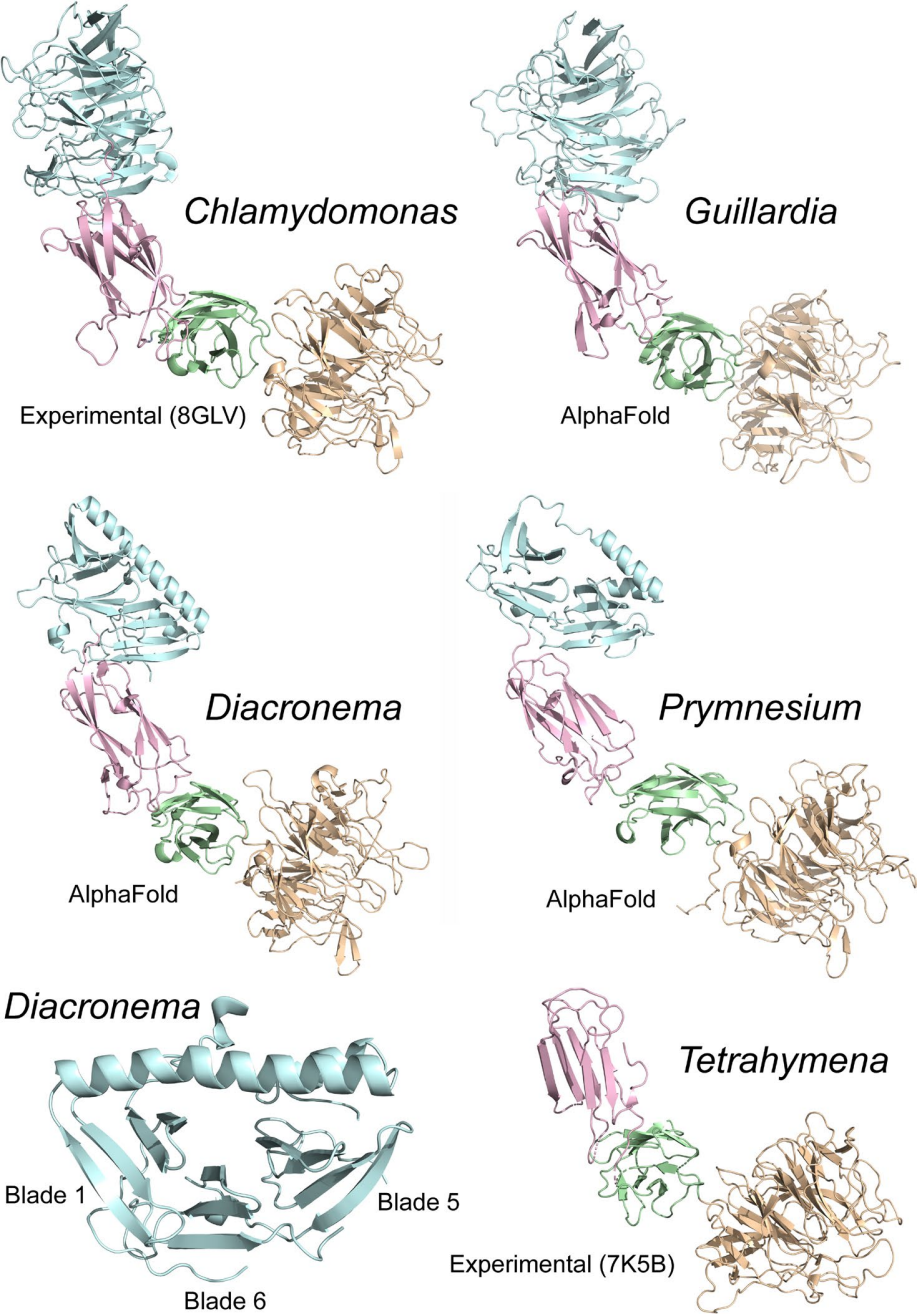
Divergence of the N‐terminal domains of outer arm dynein α heavy chain orthologues. Ribbon diagrams of the N‐terminal regions of the third outer arm HC motor from four supergroups. Three distinct arrangements are clearly present. All contain a kelch repeat β‐propeller at the very N‐terminus followed by two Ig domains. In *Tetrahymena* (SAR), this then connects directly to the motor domain. However, in other Diaphoretickes, there is an additional kelch repeat‐based β‐propeller structure. In *Chlamydomonas* and *Guillardia*, this second propeller contains the standard six blades, with blades 1–5 formed from four β strands and with blade 6 having only three strands. However, in haptophytes (*Diacronema* and *Prymnesium* are shown) blades 2, 3, and 4 have been lost entirely and replaced by a single helix that packs across the half propeller formed by blades 1, 5, and 6. An enlargement of the partial propeller from *Diaconema* is shown at *bottom left*. The color code is wheat (β‐propeller ^#^1), pale green (Ig ^#^1), light pink (Ig ^#^2) and pale cyan (β‐propeller ^#^2). Large unstructured loops have been omitted for clarity—see Figure 5 for their location.

**FIGURE 5:**
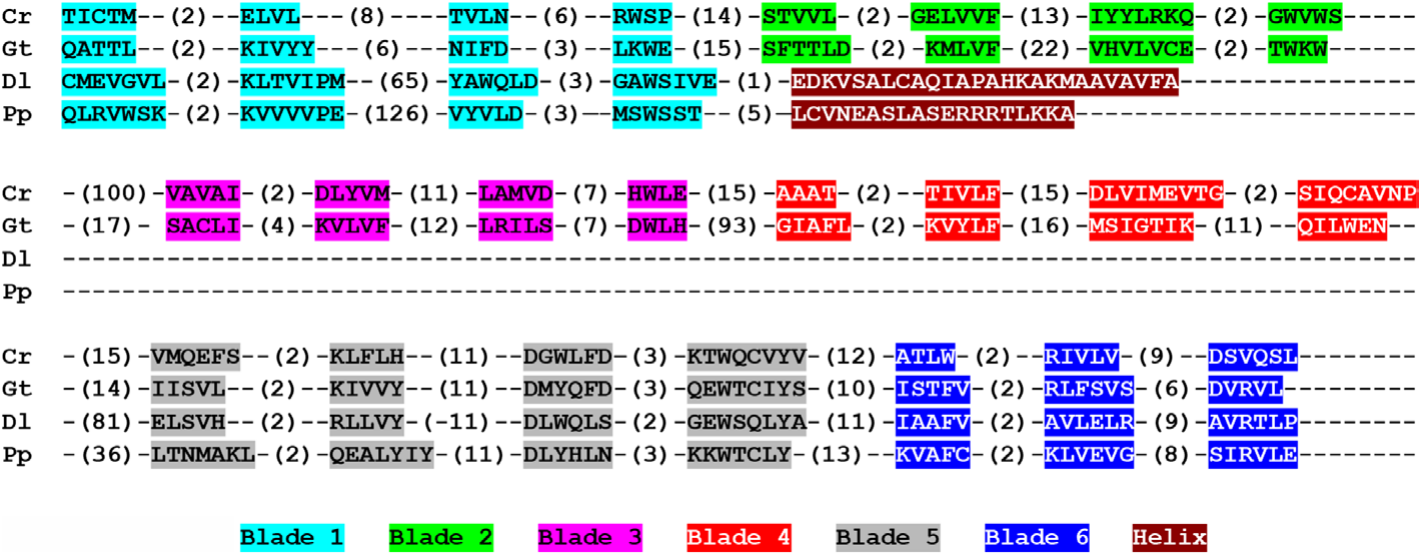
Alignment of secondary structural elements in the second β‐propeller. The secondary structure elements comprising the second full or partial β‐propeller domains from *Chlamydomonas* (Cr), *Guillardia* (Gt), *Diacronema* (Dl), and *Prymnesium* (Pp) were identified from the experimental *Chlamydomonas* structure (8GLV) and AlphaFold models. (n) represents the number of residues between the individual elements. Note that there are large unstructured loops between blades 2 and 3 (*Chlamydomonas*), 3 and 4 (*Guillardia*) and within blade 1 between strands 2 and 3 for both haptophytes (*Diacronema* and *Prymnesium*). In haptophytes, the entirety of blades 2, 3, and 4 have been replaced by another large insertion containing a single α‐helix that packs against the remaining blades.

Analysis of *Chlamydomonas* α HC orthologs within the Diaphoretickes revealed that some sequences contain gaps of varying size within the N‐terminal region while others do not. Subsequent sequence alignments found that an additional N‐terminal sub‐domain is present in chlorophyte, cryptophyte, and haptophyte orthologs, but absent from those in the SAR. In both *Chlamydomonas* (this domain is present in the experimental cryo‐EM structure, PDB 8GLV; Walton et al. 2023) and the cryptophyte *Guillardia theta* (XP_005834820; based on AlphaFold modeling), this additional domain contains kelch repeats forming the six blades of another β‐propeller structure with an extended unstructured loop region (Figures 4 and 5). Oddly, only part of this extra sequence is present in the ortholog from the haptophyte *Prymnesium parvum* (KAL1521978) and AlphaFold modeling predicts that what remains would form only half of a β‐propeller structure comprising blades 1, 5, and 6. At first glance, this seemed rather unlikely to represent a stable folded entity and raised the possibility that this haptophyte sequence might be incomplete. However, BLAST searches with the *Prymnesium* sequence returned additional haptophyte orthologs, for example, KAG8462790 from *Diacronema lutheri* and KOO23494 from *Chysochromulina tobinii*. These also had similar sequence gaps, and AlphaFold modeling again suggests they retain only a partial second β‐propeller. These haptophyte sequence data were generated and assembled by different groups several years apart from samples obtained from a Norwegian fjord (*Diacronema*), a fresh‐water creek in the Colorado River basin (*Prymnesium*), and a strain deposited many years previously at the National Center for Marine Algae (*Chysochromulina*). Thus, it seems unlikely that the loss of this region is a consequence of some systematic sequence assembly error or other artifact but rather reflects a further diversification of this domain specifically in haptophytes.

In these second haptophyte β‐propeller modules, a large unstructured loop region is inserted between strands 2 and 3 of blade 1, and a second large insertion occurs between strand 4 of blade 1 and strand 1 of blade 5. AlphaFold modeling indicates that this second insertion includes an α helix that is predicted to pack against the exposed flat face of the half propeller that is formed from blades 1, 5, and 6, potentially acting to stabilize it. All strands forming blades 2, 3, and 4 are absent (Figures 4 and 5). Notwithstanding this peculiarity, it is clear that an additional domain is present in the sub‐N‐terminal region of α dynein HCs from Chlorophyta, Cryptista, and Haptophyta. Thus, in these groups, the N‐terminal HC segment consists of a β‐propeller that interacts with the outer arm β HC, followed by two Ig domains, and then a second full or partial β‐propeller before joining a standard dynein motor domain. The specific lack of the second β‐propeller in Alveolata is confirmed by the absence of related sequences in all examined members of this clade and indeed of an equivalent structural domain in the high‐resolution cryo‐EM reconstruction of *Tetrahymena* outer arm dynein (PDB: 7K5B). Although similar experimental data do not currently exist for any members of the Stramenopila or Rhizaria, to date, no dynein from members of either group has been identified that contains an appropriate sequence motif.

There is some controversy over the precise inter‐relationships of supergroups within the Diaphoretickes, with two recent phylogenies splitting SAR and Haptophyta from Archaeplastida and Cryptista (Tikhonenkov et al. 2022; Torruella et al. 2025) rather than the arrangement suggested by (Williamson et al. 2025) in which SAR diverges first from the other groups (see Figure 3). Thus, when this additional β‐propeller domain originated is uncertain, but there are two basic possibilities. It may have been present in the common ancestor of all Diaphoretickes and subsequently lost only in the lineage that gave rise to SAR. Alternatively, it might have been absent in the Diaphoreticke common ancestor and gained in the lineage that led to Chlorophyta, Cryptista, and Haptophyta, where it was subsequently further modified.

What role this additional domain plays and what selective pressure drove its' inclusion is unclear. However, the organization of HCs in the 96‐nm axonemal repeat is such that this intriguing α HC domain is closely apposed to its own AAA ring (Figure 2). Potentially then it might bind across AAA2 and AAA3 during power stroke transitions of the AAA ring, which is reminiscent of the interactions of Lis1 with the cytoplasmic dynein motor unit that allows for processive motility under high load (Reddy et al. 2016).

## 4 Functional Significance of the Third Outer Arm Dynein Motor

Detailed experimental data concerning the role of this third outer arm HC are available only for the *Chlamydomonas* protein, as there is a null mutant (*oda11*) (Sakakibara et al. 1991). Axonemes of *oda11* cells lack both the α HC and its tightly associated thioredoxin‐like LC (LC5) but retain the remainder of the outer dynein arm and assemble it into the axoneme. In contrast, the β and γ HC N‐terminal regions are essential for outer arm formation, and null mutants for either protein result in the complete failure of outer dynein arm assembly (Kamiya 1988; Mitchell and Brown 1994; Wilkerson et al. 1994); there are β and γ HC mutants that lack individual motor domains (*oda4‐s7* and *oda2‐t*, respectively) but which assemble dynein arms as the N‐terminal segments are retained (Liu et al. 2008; Sakakibara et al. 1993).

The *oda11* mutant swims slower than wildtype, but faster than mutants (e.g., *oda1* or *oda6*) lacking the entire outer arm (Sakakibara et al. 1991). Similarly, this strain has a ciliary beat frequency intermediate between that of wildtype and outer arm‐less strains. Both changes support the idea that the α HC provides extra power that increases the ciliary beat rate and propulsive force output of the cell. It has also been observed that when *Chlamydomonas* cells are subject to various treatments that impair swimming, such as viscous loading, redox stress, or mutational loss of axonemal subcomplexes leading to ciliary paralysis, they respond by increasing the amount of a Lis1‐like protein in the cilia (Rompolas et al. 2012). When cells under high viscous load are then washed back into normal media, an increased beat frequency is observed, suggesting that the Lis1 protein leads to higher power output. The α HC appears essential for Lis1 retention, as *oda11* mutant cilia lack detectable Lis1 protein. However, whether Lis1 acts directly to specifically modulate α HC power output remains uncertain. Furthermore, the two cilia of *Chlamydomonas* exhibit different intrinsic beat frequencies, and this property is lost in the *oda11* mutant, suggesting that the α HC is somehow involved (Sakakibara et al. 1991); recent analysis suggests that the α HC is differentially phosphorylated in the two cilia on a large loop that arches over the AAA5‐derived buttress (Sakato‐Antoku et al. 2025). Thus, the presence of this third outer arm HC is associated with both increased power output generation and the ability to impose subtle control over ciliary beating.

In conclusion, analysis of the current sequence databases confirms that the third HC found in some outer arm dyneins arose in the Diphoda within a common ancestor of the Diaphoretickes after their divergence from Discoba. Based on the phylogeny of Williamson et al. (2025), this most parsimoniously involved replacement of a standard HC dimerization interface on a duplicated β HC with a different N‐terminal segment consisting of a β‐propeller and two Ig domains as now found in members of SAR. This novel adaptation underwent further diversification in the lineage leading to Archaeplastida, Cryptista, and Haptophyta gaining a second β‐propeller domain, which was then further modified in the ancestor of haptophytes. However, it is important to note that two other recently recovered phylogenies for the Diaphoretickes split Archaeplastida and Cryptista from SAR and Haptophyta (Tikhonenkov et al. 2022; Torruella et al. 2025) which, if correct, would imply that the second β‐propeller is ancestral and that its modification and loss in Haptophyta/SAR occurred subsequent to the divergence from Archaeplastida/Cryptista. Given the location of this second β‐propeller directly adjacent to the AAA motor unit, it will be fascinating to manipulate these variant domains to assess their roles in dynein biology and their potential for impacting motor activity.

## 5 Bioinformatics and Structure Display

Orthologues of the *Chlamydomonas* outer arm α HC were identified using BLASTP searches at the National Center for Biotechnology Information (NCBI; Table 1). To be included, a hit had to contain both a clear dynein motor domain signature and an N‐terminal β‐propeller + Ig domain structure. Sequence alignments were generated using CLUSTALW. For *Chlamydomonas* and *Tetrahymena*, the experimental cryo‐EM structures (8GLV and 7K5B, respectively) were used to analyze the N‐terminal domains. For all other organisms, these regions were modeled using AlphaFold 3 (Abramson et al. 2024). Structures were displayed using the PyMOL molecular graphics system v.2.4.0 (Schrödinger LLC).

## Nomenclature

The naming of dynein components is both complex and confusing. The root cause of this dates to the original biochemical purifications of these motors when proteins were named based on their migration patterns in SDS gels before the underlying sequences were known. Consequently, in many cases, the same name was given to non‐orthologous components in different organisms due to variations in gel migration patterns and/or the number of subunits. Here I use the nomenclature from *Chlamydomonas*. See Hom et al. (2011) and Braschi et al. (2022) for correspondence to other naming schemes.

## Conflicts of Interest

The author declares no conflicts of interest.

## Data Availability

All sequence data used in the preparation of this review are freely available at the National Center for Biotechnology Information. Experimental cryo‐EM structures for the Chlamydomonas 96‐nm axonemal repeat unit (8GLV) and Tetrahymena outer arm dynein (7K5B) can be downloaded from the Protein Data Bank.
